# Survey of farmers’ knowledge of cassava mosaic disease and their preferences for cassava cultivars in three agro-ecological zones in Benin

**DOI:** 10.1186/s13002-018-0228-5

**Published:** 2018-04-25

**Authors:** Jerome Anani Houngue, Justin S. Pita, Gilles Habib Todjro Cacaï, Martine Zandjanakou-Tachin, Emmanuel A. E. Abidjo, Corneille Ahanhanzo

**Affiliations:** 1 Central Laboratory of Plant Biotechnology and Plant Breeding, Department of Genetics and Biotechnology, Faculty of Science and Technique, University of Abomey – Calavi, Abomey-Calavi, Benin; 2 Laboratory of Plant Physiology, Université Felix Houphouët-Boigny, Abidjan, Côte d’Ivoire; 3 Laboratory of Molecular Plant Pathology, School of Horticulture and Green Space, National University of Agriculture, Porto-Novo, Benin

**Keywords:** Farmer survey, Preferred cultivars, Cassava mosaic disease, Disease severity, Disease symptoms, Staple crop, Knowledge evaluation, *Manihot esculenta*

## Abstract

**Background:**

Cassava is an important crop in Africa that is widely cultivated for its starchy tuberous root, which constitutes a major source of dietary carbohydrates. Cassava mosaic disease (CMD) is the most devastating disease affecting cassava in Africa and causes enormous losses in yield. In Benin, specifically, cultivars resistant to CMD are not commonly planted, and even when CMD is observed in fields, farmers do not implement control measures, presumably because they lack proper knowledge and training. Our study aimed to evaluate farmers’ knowledge of CMD to determine whether there is consistency between farmers’ criteria for selecting cassava cultivars and the currently CMD-recommended cassava varieties.

**Methods:**

We conducted structured interviews with 369 farmers in 20% of townships in each of three agro-ecological zones in Benin between November 2015 and February 2016. Farmers were selected randomly in each household, and their fields were assessed for CMD incidence and severity.

**Results:**

All farmers surveyed, representing a broad demographic pool with regard to education level, age group, and years of experience in cassava production, successfully recognized CMD symptoms in photos, but most (98.60%) said they did not know the causes and vectors of the disease. Most farmers (93.51%) reported that they obtain planting material from neighboring fields or their own fields. In total, 52 unique cultivars were identified, of which 3 (5.76%) were preferred based on their yield and precocity and 3 (5.76%) were preferred based on taste or ability for transformation. The assessment of disease incidence and severity showed that the areas most affected by CMD were Comè Township (37.77% of fields affected) and agro-ecological zone VIII (26.33%).

**Conclusion:**

Farmers already know how to recognize the symptoms of CMD and could implement control measures against it if they are trained by researchers. Across all surveyed areas, we identified six preferred cultivars based on the four most commonly stated preference criteria (precocity, yield, gari, and taste. Our results suggest that farmers will be more likely to use CMD-resistant cultivars and clean plant material if the plants meet their existing preference criteria. We suggest that CMD-resistant cultivars will be embraced only if the recommended cultivars are strategically aligned with the characteristics desirable to the cassava farmers in each region.

## Background

Cassava (*Manihot esculenta* Crantz) is one of the most important food crops in sub-Saharan Africa. Every part of the plant can be eaten; the starchy roots are by far the most commonly used because they are a valuable source of energy and can be boiled or processed in different ways for human consumption [1]. The leaves and tender shoots are a rich source of proteins and vitamins and are consumed as a vegetable in many regions [2]. Two advantages of cassava compared with other food crops are the flexibility in its planting and harvest times and its tolerance to drought [3]; it can survive and produce high yields in conditions that cereal crops would fail [4]. In Benin, cassava is the second most commonly grown crop after maize. Cassava is the most important crop in multi-crop systems, it is found in a wide range of markets, and it provides a stable source of income and food; thus, it is a form of food security for many households.

Cassava mosaic disease (CMD) is the most devastating cassava disease in Africa, causing annual yield losses of 12–23 million tons, which is equivalent to $1.2 to 2.3 billion USD [5]. CMD is caused by cassava mosaic geminiviruses (family *Geminiviridae*, genus *Begomovirus*), which are transmitted by the whitefly vector, *Bemisia tabaci* (order Hemiptera, family Aleyrodidae), and through the use of infected cuttings during vegetative propagation [6–8]. CMD symptoms are present in most cassava fields, and the disease affects many cultivars in Benin. Farmers are urged to adopt resistant varieties and cultivars, but successful adoption is dependent on the presence of preferred traits among the available cultivars. Sometimes, the recommended varieties are rejected by farmers because they do not correspond with farmers’ preferences. Preferences such as taste, yield, and transformation ability of cassava have typically been ignored when screening cassava germplasm for CMD resistance [9]. We predicted that the failure to integrate CMD-resistant cassava cultivars in Benin is the result of the characters not matching the characteristics that are most desirable to small-scale farmers. We further predicted that the reason CMD is not controlled properly in Benin is that small-scale farmers lack proper knowledge and training. (For example, we have previously observed that some farmers in this region think that they can control the virus using pesticides.) In this study, we conducted a large-scale survey in Benin to gather vital information about farmers’ preferences for cassava cultivars and their knowledge of CMD. This participatory approach aimed to reveal the potential social constraints to the adoption of CMD-resistant cassava varieties [8, 10] in Benin. The study’s specific aims were as follows: (1) to evaluate famers’ knowledge of CMD, (2) identify the local cassava varieties being cultivated by farmers, and (3) understand the cultivar characteristics that farmers prefer. Knowledge about how viruses are transmitted and their infection cycle is important to control the spread of the disease, as no approved or reliable antiviral products are generally available. When managed poorly, CMD and other viruses can cause a complete loss of cassava yields; it is therefore important to understand what farmers know about CMD, their perceptions about how infection affects cassava yields, how they currently manage the disease, their criteria for selecting cassava cultivars, and how they source planting materials. Building knowledge among farmers is probably the most important strategy for controlling CMD, and the first step in building this knowledge is to understand the current state of farmers’ knowledge.

## Methods

### Areas surveyed

**Fig. 1:**
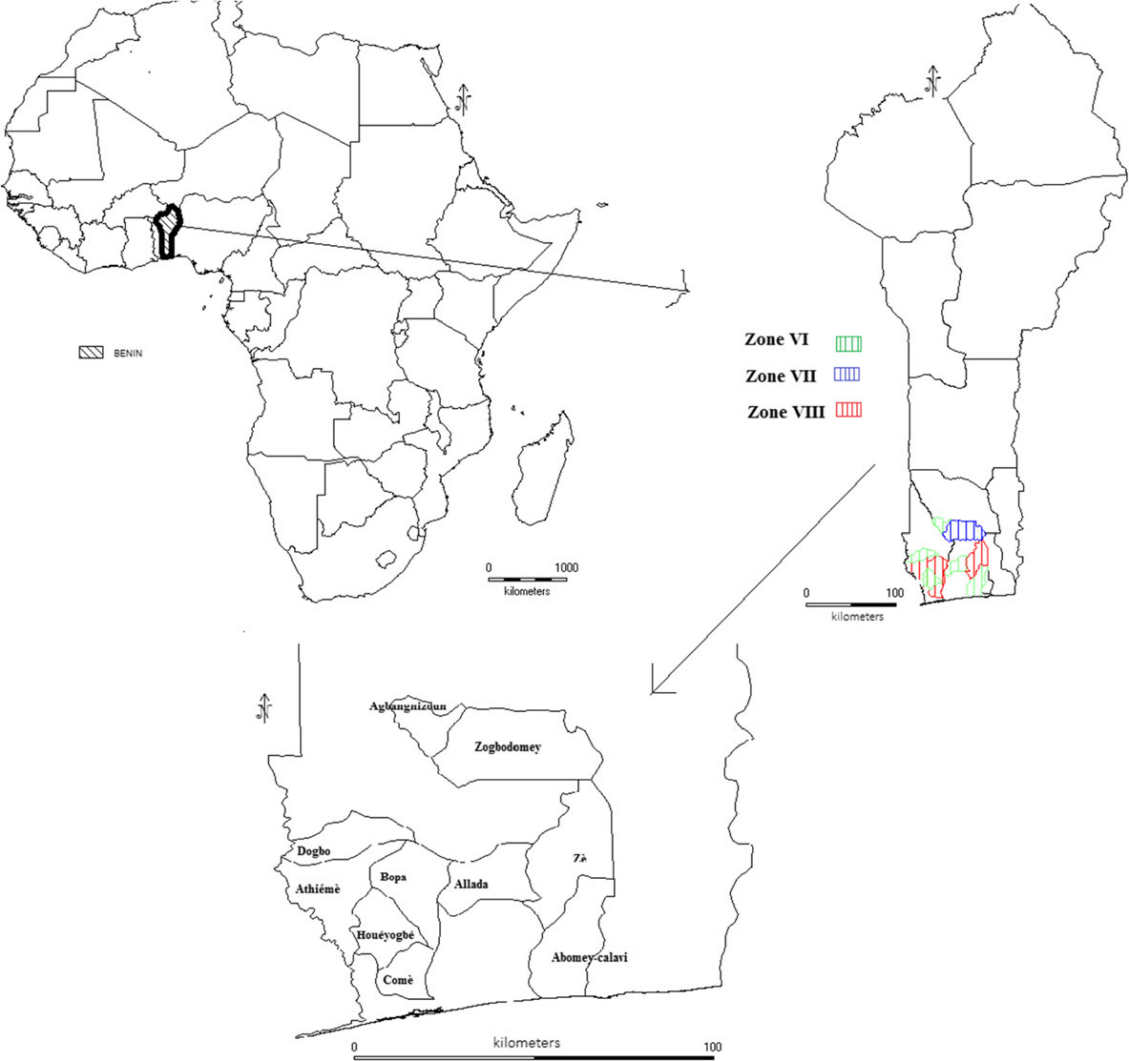
Map of survey areas in three agro-ecological zones in Benin

The study was conducted in three agro-ecological zones (AEZ) in Benin: AEZ VI (Zone of Bar Land), AEZ VII (Zone of Fishery), and AEZ VIII (Zone of Depression) where cassava is either the primary crop or the secondary crop (after maize) [11]. We selected five townships from AEZ VI (Houéyogbé, Abomey-calavi, Dogbo-tota, and Zè, Agbangnizoun), three townships from AEZ VII (Come, Bopa, and Athieme), and one from from AEZ VIII (Zogbodomè) (Fig. 1). These areas are characterized by a Sudano–Guinean climate and have a bimodal rainfall pattern between 800 and 1400 mm annually, with two distinct rainy seasons and two dry periods.

### Sampling and data collection

Information on farmers’ knowledge of CMD was collected using semi-structured questionnaires; the state of cultivars in their fields was assessed by conducting field observations. A multi-stage sampling method was used to select farmers to participate to interviews. In the first stage, we selected 20% of the townships in each AEZ based on their relative importance in cassava production. Five townships were selected in AEZ VI, three in AEZ VII, and one in AEZ VIII. In the second stage, we randomly sampled three villages per township, for a total of 27 villages (Fig. 1). In the third stage, we randomly sampled 15 to 20 households per village. The sample size (total number of surveys) was determined by applying the Dagnelie formula [12]:

$$ N=\frac{\ {\bigcup}_{1-\alpha /2}^2\ast P\left(1-P\right)}{d^2} $$

where *N* is the sample size; U_1−ᾳ/2_ is the reduced centered variable number = 1.96; *P* is the proportion of interviewed farmers who recognized CMD symptoms on photos according to what they saw in their farm; and *d* is the margin of error set at 5%. The application of this formula gives *N* = 369 farmers, of which 219 were in AEZ VI, 105 were in AEZ VII, and 45 were in AEZ VIII (Table 1).

**Table 1:** Sample size (number of farmers surveyed) by agro-ecological zone

Agro-ecological zone (AEZ)	Sample size
AEZVI	219
AEZ VIII	45
AEZ VII	105
Total	369

The identification of responding households was facilitated by rural development officials (RDRs) and sometimes by village leaders. The questionnaires were tested during a pre-survey on 30 farmers in three AEZ, which allowed the research team to optimize their techniques to survey the farmers. Two kinds of questions were asked to farmers: closed (yes/no) questions and open-ended questions that gave farmers the freedom to give their opinions [13]. All interviews were conducted in the local language of the villages (Fon, Mina, Sahouè, Adja, and Nago) to encourage farmers to explain confidently their views. The assessment of farmers’ knowledge of CMD was done with photographs of plants exhibiting CMD symptoms or infested with insect pests as described by Chikoti et al. [8]. Some questions in the questionnaire were rephrased to enable farmers to better understand and respond as fully as possible. To evaluate farmers’ criteria in their selection of cassava cultivars, we used the survey method described by Dansi et al. [14]. Briefly, farmers were asked to list the characteristics that a cassava cultivar must have in order to be widely adopted in their village. They were then asked to rank these selection criteria according to their importance.

### Incidence and severity of CMD

Field surveys were carried out in nine townships to establish the correlation between farmers’ knowledge of CMD and the incidence and severity of CMD in their township. For this purpose, three cassava fields containing plants grown for 3 months at least 15 km apart [15] were surveyed in each township. We selected and scored 30 plants along two diagonals of the field using the CMD scale described by Hahn et al. [16]. Disease incidence was determined by dividing the number of infected plants by the total number of observed plants. Classes of disease severity were constructed based on the Hahn scale [16], and the townships were grouped according to these classes using principal component analysis (PCA).

### Analysis and data processing

Survey data was processed with Sphinx Statistical Processing Software (version 4.5). Farmers were grouped according to five categories: socio-cultural group, age, education level, years of experience growing cassava, and gender (Table 2). A frequency distribution was plotted to compare the response rates for each category. Chi-squared tests were used to test for relationships between farmers’ knowledge of CMD and the five categories. Separate chi-squared tests were used to test whether a farmer’s preferences for a particular cultivar were related to the region in which they lived and whether these characteristics were present in their preferred cultivars.

**Table 2:** Socio-demographic characteristics of respondents

Socio-demographic characteristics	Modality	Percentages
Experience in production	≤ 15 years	0.3
	15–30 years	33.5
	> 30 years	66.2
Sex	Male	62.4
	Female	37.6
Age	≤ 15 years	0
	15–30 years	5.9
	> 30 years	94.1
Marital status	Married	96.5
	Single	3
	Widow(er)	0.6
Education	No schooling	74.9
	Primary	10.3
	Secondary	11.6
	Tertiary	3.2

The most commonly grown cultivars were considered “preferred,” and we ran a correspondence analysis (CA) to group preferred cultivars by criterion of choice. Disease incidence and severity were analyzed using Excel and Minitab 16 (version 2010). CMD incidences were expressed as percentages and values were arcsine-transformed [17] to facilitate logistic regression. We performed a clustering analysis using multiple correspondence analysis (MCA) to group townships by mean severity class.

## Results

### Socio-demographic characteristics of surveyed farmers

Table 2 provides the socio-demographic characteristics of the 369 farmers surveyed. All the farmers surveyed were cassava famers. Most farmers surveyed were male (62.40%). The majority of farmers (94.10%) were ≥ 30 years old and most (96.50%) were married. Most cassava farmers (74.90%) had no schooling. The majority (66.20%) of farmers reported that they have had at least 30 years of experience in cassava production. This broad representation suggests that our random sampling method accounts for the demographics in these areas.

### Recognition of cassava mosaic disease

All 369 farmers were able to recognize CMD in the photos provided during the survey. A chi-squared test suggested that recognition of CMD symptoms did not depend on a farmer’s age, gender, education, or years of experience with cassava (Table 3). However, 98.60% of farmers reported that they did not know the causes and vectors of CMD. Most (62.43%) farmers said that they believe CMD reduces yields; 37.57% reported that they believe, based on their observations in their fields, that CMD prevents cassava from rooting.

**Table 3:** Chi-squared tests to investigate whether farmers’ knowledge of CMD differed by socio-demographic factors

Factors	*χ* ^2^	ddl	*P*
Sex	1.65	1	0.20 NS
Age	0.89	1	0.45 NS
Experience	0.10	2	0.96 NS
Education	3.64	3	0.30 NS

*ddl* degree of liberty, *P* probability, *NS* not significant at 5% threshold

### Origins of plantation material and farmer action against CMD

**Fig. 2:**
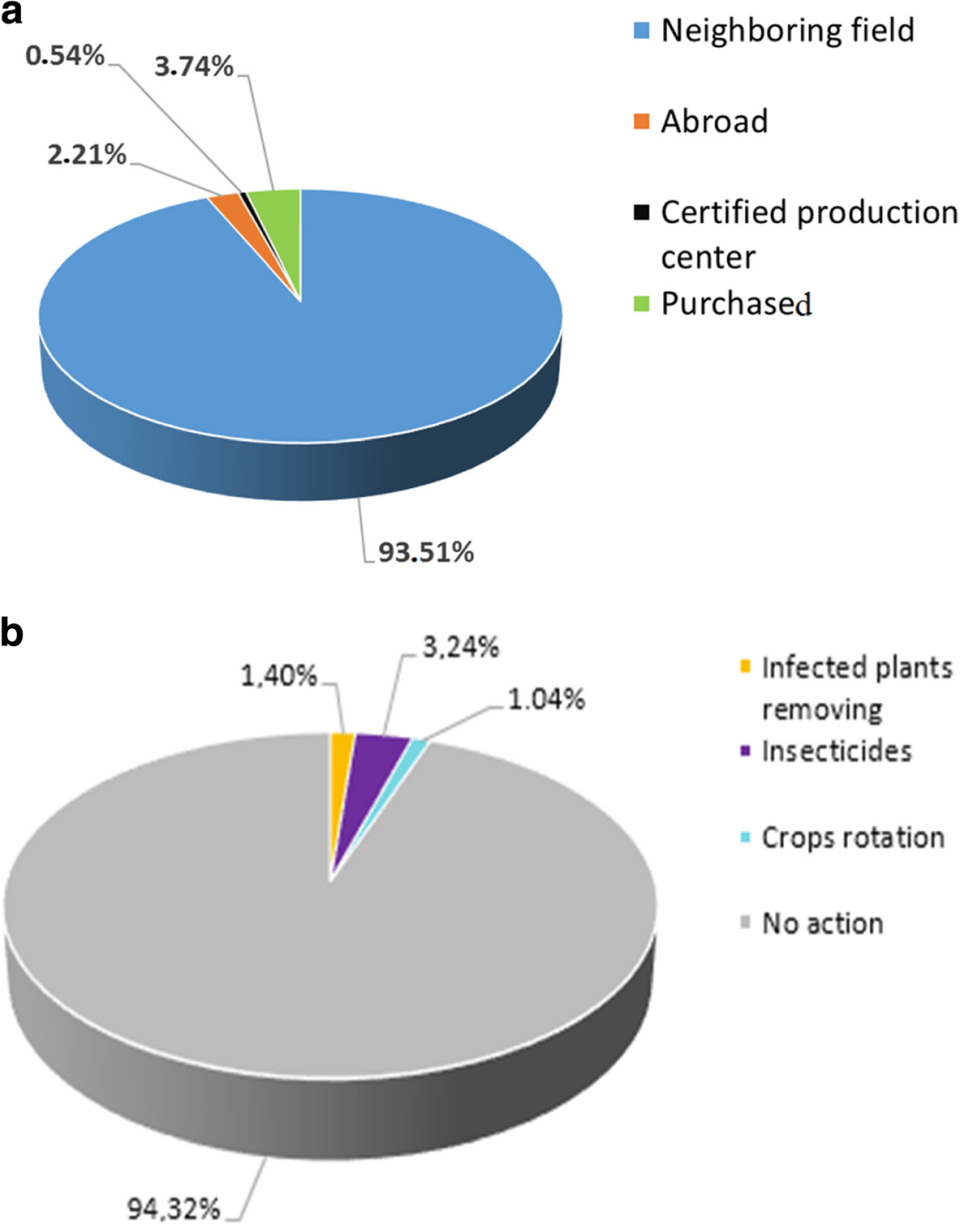
Origins of cassava planting material reported by 369 surveyed farmers in Benin. **a** Origins of planting material, **b** disease management practices

Almost all of the 369 farmers surveyed (93.51%) reported that they obtain planting material from neighboring fields and use the same material over many seasons. A minority of farmers (0.54%) acquired planting material from research centers, such as the National Institute of Agricultural Research of Benin (INRAB) and the Regional Center of Agricultural and Rural Development (CARDER) or from abroad (Fig. 2a). Analysis of independence revealed that the source of planting material was significantly related to AEZ (*χ*^2^ = 49.32, *P* = 0.001).

A lack of CMD control measures was noted through the study. Only 1.4% of interviewed farmers took action to destroy plants infected with CMD (Fig. 2b). Only 3.2% of farmers used insecticides to control insects such as whitefly and mites (Fig. 2b). In contrast, we found that of the 21 farmers who had been sensitized to CMD, 5 took action to destroy infected plants.

### Criteria for selection of cassava cultivars

**Fig. 3:**
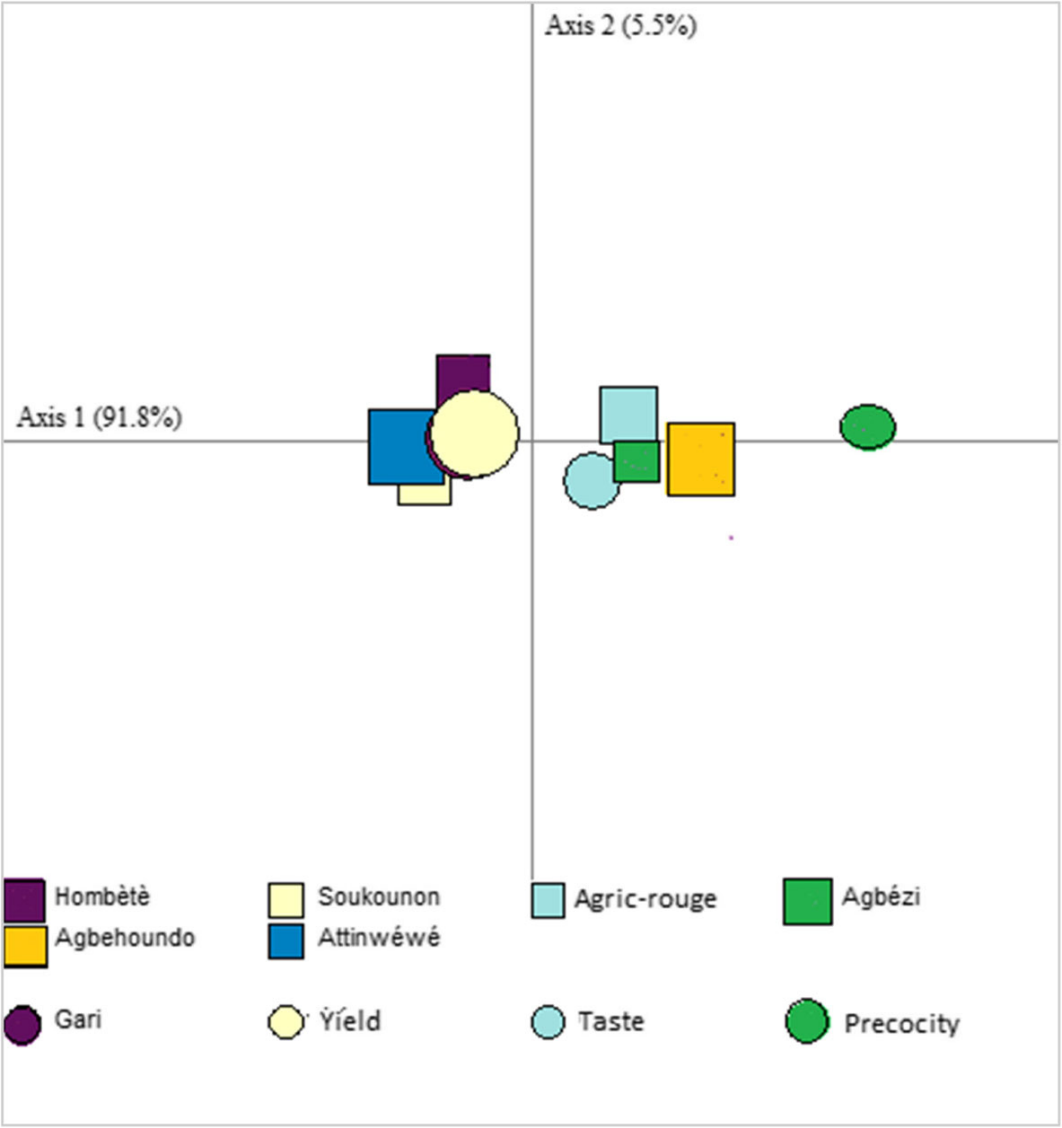
Correspondence analysis grouping of preferred cultivars by township and farmers’ four most important criteria (gari, yield, taste, and precocity)

We recorded 52 unique cultivars (Table 4), of which 6 were preferred cultivars by at least 50% of the farmers (Table 5). The survey revealed that farmers prioritize the following characteristics when selecting cassava cultivars: yield, the quality of products resulting from the processing of cassava (gari, ethanol), the precocity of the cultivar, and taste (organoleptic quality). A chi-squared test showed that a farmer’s preferred characteristics for cassava cultivars were significantly related to the AEZ in which he/she lived (*χ*^2^ = 58.84, ddl = 8, *P* = 0.01%). A separate chi-squared test showed that a farmer’s preferred characteristics were also significantly related to a farmer’s preferred cultivar (*χ*^2^ = 129.09, ddl = 68, *P* = 0.01). This dependence between choice criteria and preferred cultivars is explained by the CA (Fig. 3). The inertia of two axes (97.3%) shows that the CA summarizes all factors expressed. Soukounon, Atinwéwé, and Agbézi cultivars were preferred because of yield and early maturity, whereas Agric-rouge, Hombètè, and Agblehoundo cultivars were preferred because of their taste and gari (Fig. 3).

**Table 4:** Cultivars of cassava and the villages in which they are cultivated

Cultivars	Villages
Atinvovo	Niaouli, Nouzounkpa, Agbodjédo, Agrimey, Kotto, Koui, Ayou-centre, Haindé
Hombètè	Hounssagodo, Yokpodjèvié, Foncomè, Soukpotomé, Haindé, Atchannou, Lizèmè, Tokpadji, Hècondji, Médémahoué, Tokpoé, Houéglé, Comè, Honvè, Ayou-centre, Agbodjèdo, Niaouli, Nouzounkpa
Ahôtonon	Dessounkpa, Lamè, Agrimey, Houéglé, Foncomè
Awonliton	Ayou-centre
Rb	Ayou-centre
Ben	Ayou-centre
Soukounon	Dovota, Dessounkpa, Lamè
Adjatindaho	Agbodjedo, Koui
Illakè	Nouzounkpa, Yokpodjèvié, Hounssagodo
Sèguè	Yokpodjèvié, Nouzounkpa, Hounssagodo, Tokpoé
Dossi	Ayou-centre, Hounssagodo
Globokouté	Hounssagodo
Bassi	Yokpodjèvié, Hounssagodo
Limouti	Hounssagodo, Ayou-centre
Agric-rouge	Honvè, Comè, Atadocomè, Houéglé, Avègodo, Tokpoé, Massè, Lizèmè, Atchannou, Haindé, Hècondji, Tokpoé, Foncomè, Gouhoun, Honton, Soukpotomè, Médémahoué
Sowé	Dovota, Lamè, Dessounkpa, Koui
Carder	Nouzoukpa
Bonoua	Agrimey
Gbégo	Agrimey, Koui
Gbézé	Agrimey, Foncomè
Dodo	Agrimey, Koui
Alaboukoun	Koui
Odohoungbo	Haindé
Dogué	Koui
Doukoui	Agrimey, Koui
Gologoun	Nouzounkpa, Hounssagodo, Yokpodjèvié
Kpèkè	Agbodjèdo
Flèkè	Niaouli, Agbodjèdo, Yokpodjèvié, Hounssagodo, Ayou-centre
Hata	Honvè, Soukpotomè, Lizèmè
Agblehoundo	Honvè, Comè, Tokpoé, Gouhoun, Honton, Soukpotomè, Médémahoué, Haindé, Hècondji, Tokpadji, Massè, Lizèmè, Soukpotomè
Yovovi	Honton
Agbézi	Foncomè, Gouhoun, Honton
Ahôtonongbadji	Foncomè, Honton
Gbendokoutouti	Foncomè
Abatouin	Honton, Foncomè
Houèton-kotou	Nouzounkpa, Niaouli, Agbodjèdo, Hounssagodo
Atinwéwé	Agrimey, Kotto, Ayou, Haindé
Codjovi	Tokpoé, Massè, Tokpadji
Gbakaya	Houéglé, Avègodomè, Tokpoé, Tokpadji, Massè
Globo	Tokpoé, Massè
Sikuwé	Tokpoé
Ahokpo	Atadomè, Avègodomè, Atchannou
Gaciagamè	Atadomè, Houéglé, Avègodomè, Haindé
Acrakouté	Haindé
Koyinvo	Comè
Détahouboto	Foncomè
Zannou	Atadomè
Ouémènou	Atadomè, Médémahoué, Haindé
Doubokou	Agrimey, Koui
Gimgbo	Atadomè
Kalaba	Houéglé
Houla	Agrimey

**Table 5:** Preferred cultivars by township

Townships	Number of cultivars	Preferred cultivars
Zè	13	***Hombètè, **Atinwéwé
Allada	13	***Atinwéwé
Agbangnizoun	3	***Soukounon
Zogbodomey	13	***Atinwéwé
Dogbo	13	***Agbézi, **Agric-rouge
Bopa	10	***Agblehoundo
Athiémé	9	***Agric-rouge
Houéyogbé	12	***Agric-rouge, **Agblehoundo
Comè	8	***Agblehoundo, **Agric-rouge

***Highly preferred (cited as preferred by at least 75% of farmers in township); ** moderately preferred (cited as preferred by at least 50% of farmers in township)

### Incidence of CMD

**Fig. 4:**
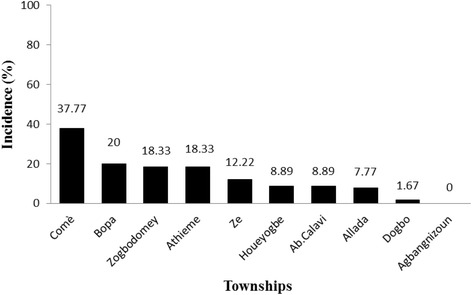
Incidence of cassava mosaic disease by township in Benin

**Fig. 5:**
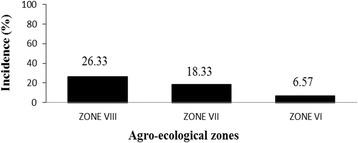
Incidence of cassava mosaic disease by agro-ecological zone in Benin

The assessment of CMD incidence by township showed that Comè was the most affected township (37.77%) followed by Bopa (20%), Athiémé and Zogbodomey (18.33%), Zè (12.22%), Abomey-Calavi and Houéyogbé (8.89%), Allada (7.77%), Dogbo (1.67%), and Agbangnizoun (no reported cases). Among the three AEZ studied, disease incidence was highest in AEZ VIII (26.33%) followed by AEZ VII (18.33%) and AEZ VI (6.57%), (Figs. 4 and 5). Chi-squared tests showed that differences in the distribution of CMD were more significant by AEZ than by township (*P* < 0.001) (Table 6). Therefore, sampling in AEZ explained better the distribution of CMD in the study area. Binary logistic regression (Table 7) showed that disease incidence in AEZ VIII, AEZ VI, and Comè township was significantly higher than in AEZ VII and the other townships (*P* < 0.001).

**Table 6:** Chi-squared test of the distribution of CMD

	ddl	Log-	*χ* ^2^	*P*
Ord. Orig.	1	− 330.91		
Agro-ecological zone	2	− 313.35	35.11	2.37E−08
Township	6	− 298.81	29.08	5.87E−05
Zone*township	0	−298.81	0.00	3.00E+30

* means interaction

**Table 7:** Logistic regression by township and agro-ecological zone

	Level	Column	Estimation	Standard	Wald	*P*
Ord. Orig		1	−7.05	0.57	151.05	0.00
Zone	Zone VI	2	4.72	0.86	30.11	0.00
Zone	Zone VIII	3	5.08	0.71	51.41	0.00
Township	Houeyogbe	5	0.00	0.52	0.00	1.00
Township	Dogbo	6	−1.75	1.07	2.65	0.10
Township	Allada	7	−0.15	0.54	0.07	0.79
Township	Zè	8	0.36	0.49	0.53	0.47
Township	Comè	10	1.47	0.39	14.38	0.00
Township	Bopa	11	0.59	0.39	2.20	0.14

### Severity of CMD

**Fig. 6:**
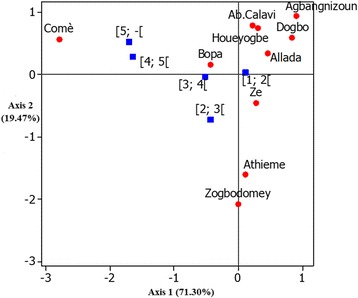
Townships in Benin clustered by disease severity score for cassava mosaic disease. Blue squares denote disease severity classes. Red squares denote townships

Townships differed significantly (*P* < 0.001) in disease severity (Table 8). We used MCA to group townships by mean severity class: group A was townships dominated by severity score 1 (healthy plants), group B was townships dominated by severity score 2, group BC was townships dominated by severity score 3–4, and group C was townships dominated by severity score 5 (most diseased). Disease severity was highest in Comè and Bopa (Fig. 6). The townships Houéyogbé, Athiémé, Abomey-Calavi, Zè, Zogbodomey, and Agbangnizoun were less affected compared to Comè. MCA showed that the townships of Agbangnizoun, Dogbo, Zè, and Allada and the disease severity class [1; 2[were positively correlated to axis 1 in contrast to townships of Comè, Bopa and disease severity class [4; 5[, which were negatively correlated. Houéyogbé, Athiémé and Abomey-Calavi with disease severity class [2, 3[were positively correlated to axis 2 (Fig. 6).

**Table 8:** Clustering of townships by class or degree of severity of CMD

Classes	Size	Means	Groups	*P*
[1,2[	10	1.59	A	0.00***
[2,3[	10	0.41	B	
[3,4[	10	0.36	BC	
[4,5[	10	0.20	BC	
[5,- [	10	0.13	C	

*** very high significant, ** high significant, * significant

## Discussion

The results of this study showed that farmers in Benin can identify CMD by photographs because they have seen the disease in their fields and know that these symptoms constitute diseased plants. However, Benin farmers generally lack critical knowledge about CMD, its transmitting vectors, and how it can be controlled. We believe this lack of knowledge constitutes the major obstacle for CMD control in Benin. Farmers who were sensitized and trained to identify the manifestations of the disease could differentiate diseased and healthy plants. These few farmers who were familiar with CMD reported that they were sensitized to it by rural development agents. Our survey results suggests that farmers could be sensitized and trained by researchers and farming agents on the symptoms of CMD, thus giving them the ability to take proper action to control the disease in their own fields and minimize its spread to neighboring fields. These observations were in accordance with those obtained by Chikoti et al. [8], who also found that the lack of CMD knowledge by farmers requires researchers and extension agents to sensitize and train farmers. In our study, we found that the few farmers who had been sensitized to CMD took action to destroy infected plants and used insecticides to control whitefly, mites, and other insects. Thus, we recommend that training be widely implemented in CMD-prone areas to give these farmers the knowledge and tools to take control measures as soon as the disease is identified.

Our study identified cultivars in each AEZ that are preferred by farmers, which provides an opportunity for these preferred cultivars to be screened for CMD resistance. The farmers generally preferred the precocious cultivars with good taste, high yields, good gari, and alcohol production. These observations are in agreement with those made by Njukwe et al. [18] who evaluated the farmers’ preferred characteristics of cassava in Cameroon.

Most farmers used planting materials from previous seasons or nearby fields. Consequently, they frequently replanted infected cuttings. CMD is a systemic infection; once it infects a cassava plant, it proliferates and all plants from its infected cuttings will be diseased. Using infected cuttings from previous seasons also increases the severity of the disease and favors the rapid expansion of the disease [19]. Farmers who are unaware that their planting material is infected further spread CMD by sharing cuttings with their neighbors. These farmers do not realize the impact of CMD on cassava yields nor do they recognize that neighboring (non-cassava) crops can harbor whitefly; thus, these whitefly-infested fields go untreated, and CMD is further spread from one field to another.

## Conclusion

This study established that farmers in the three major AEZ of Benin already know how to recognize the symptoms of CMD but do not realize how CMD could affect cassava yields or how it could spread through the use of infected cuttings or the whitefly vector. We recommend that farmers receive training by researchers on the disease so that farmers can implement adequate control measures when CMD is identified. Across all surveyed areas, we identified six preferred cultivars based on the four most commonly stated preference criteria (precocity, yield, gari, and taste). Our results suggest that farmers will be more likely to use CMD-resistant cultivars and clean plant material if those cultivars meet farmers’ existing preference criteria. This constitutes the best long-term solution to CMD, because if farmers use and disseminate CMD-resistant cultivars, yield losses will be reduced even if farmers lack knowledge about the disease. Thus, to ensure that CMD-resistant cultivars are integrated, we suggest that cultivar recommendations be strategically aligned with the characteristics desirable to cassava farmers in each region.

## Abbreviations

AEZ: Agro-ecological zones
CARDER: Centre d’Action Régional pour le Développement Rural
CMD: Cassava mosaic disease
INRAB: Institut National de recherche Agricole du Benin
WAVE: West African Virus Epidemiology
